# The role of the gut microbiome and its metabolites in metabolic diseases

**DOI:** 10.1007/s13238-020-00814-7

**Published:** 2020-12-21

**Authors:** Jiayu Wu, Kai Wang, Xuemei Wang, Yanli Pang, Changtao Jiang

**Affiliations:** 1 Department of Obstetrics and Gynecology, Center for Obesity and Metabolic Disease Research, School of Basic Medical Sciences, Third Hospital, Peking University, Beijing, 100191 China; 2 Center of Basic Medical Research, Institute of Medical Innovation and Research, Peking University Third Hospital, Beijing, 100191 China; 3 Department of Physiology and Pathophysiology, School of Basic Medical Sciences, Peking University, Key Laboratory of Molecular Cardiovascular Science, Ministry of Education, Beijing, 100191 China

**Keywords:** gut microbiome, metabolism, metabolite, immune regulation, metabolic diseases

## Abstract

It is well known that an unhealthy lifestyle is a major risk factor for metabolic diseases, while in recent years, accumulating evidence has demonstrated that the gut microbiome and its metabolites also play a crucial role in the onset and development of many metabolic diseases, including obesity, type 2 diabetes, nonalcoholic fatty liver disease, cardiovascular disease and so on. Numerous microorganisms dwell in the gastrointestinal tract, which is a key interface for energy acquisition and can metabolize dietary nutrients into many bioactive substances, thus acting as a link between the gut microbiome and its host. The gut microbiome is shaped by host genetics, immune responses and dietary factors. The metabolic and immune potential of the gut microbiome determines its significance in host health and diseases. Therefore, targeting the gut microbiome and relevant metabolic pathways would be effective therapeutic treatments for many metabolic diseases in the near future. This review will summarize information about the role of the gut microbiome in organism metabolism and the relationship between gut microbiome-derived metabolites and the pathogenesis of many metabolic diseases. Furthermore, recent advances in improving metabolic diseases by regulating the gut microbiome will be discussed.

## INTRODUCTION

The worldwide prevalence of metabolic diseases, including obesity, nonalcoholic fatty liver disease (NAFLD), insulin resistance, type 2 diabetes mellitus (T2DM), atherosclerosis (AS) and polycystic ovary syndrome (PCOS), has grown dramatically (Norman et al., 2007; Popkin et al., 2012; Younossi et al., 2016; Zheng et al., 2018; Virani et al., 2020). Over the past few decades, the increasing consumption of high-calorie foods and displacement of leisure-time physical activities with sedentary activities has ultimately resulted in a positive energy balance (where energy intake exceeds energy expenditure), and these have become the main risk factors for obesity and obesity-related diseases (Heymsfield and Wadden, 2017). In this situation, the adipose tissue exceeds its ability to store all the excess energy as triglycerides, causing lipids to spill out into the circulation. This excess supplementation of lipids to nonadipose tissues, which have an impaired capacity to increase fat oxidation upon increased fatty acid availability (called metabolic flexibility), results in ectopic fat storage (Corpeleijn et al., 2009). Excessive accumulation of fat in adipocytes triggers increased production and secretion of proinflammatory adipokines, contributing to the development of insulin resistance, which is associated with the development of T2DM and NAFLD (Reilly and Saltiel, 2017). Genetically speaking, more than 99% of human genes are microbial (Gilbert et al., 2018), and microbial cells are at least as abundant as human somatic cells (Sender et al., 2016). The gut microbiome refers to the trillions of microorganisms that reside within the gut, including bacteria as well as viruses, fungi, archaea, phages and protozoa (Whitman et al., 1998), which have the capability to interact with the host in several ways. On the one hand, some gut microbiota are treated as pathogens by the host immune system, which recognizes and eliminates them. Nevertheless, the majority of the gut microbiota is nonpathogenic and symbiotic with intestinal epithelial cells. The gut microbiome plays a predominant role in nutrient metabolism upon dietary intake, xenobiotic and drug metabolism, maintenance of the structure and function of the gut barrier and gastrointestinal tract, and prevention of translocation of intestinal pathogens (Jandhyala et al., 2015). Research into the role of the gut microbiome in modulating metabolic disorders has rapidly increased over past decades. In this article, we focus on the role of the gut microbiome and its metabolites in the onset and development of many metabolic diseases, as well as the underlying mechanisms and new technology for the manufacture of a series of various target-specific drugs for therapy. We aim to provide guidance for future research in the emerging field of the gut microbiome related to the development of metabolic diseases in humans.

## THE RELEVANCE BETWEEN THE GUT MICROBIOME AND METABOLIC DISEASES

The potential role of the gut microbiome in the development of various human diseases has received considerable attention over the past decade. In particular, the gut microbiome has advanced as an important contributor to the development of many metabolic disorders such as obesity, type 2 diabetes, and nonalcoholic fatty liver disease. We summarized the changes in the gut microbiome composition in metabolic diseases (Table 1). The current global obesity epidemic is linked to lifestyle changes that are characterized by excessive energy intake and reduced physical activity. Western diet-induced obesity results in changes in the composition of the gut microbiome, such as the Mollicutes class of the Firmicutes, which were significantly increased (Turnbaugh et al., 2008). In recent years, the influence of the gut microbiome as a potential mechanism driving factors of obesity and its related comorbidity has become the focus of attention. The gut microbiota is a key interface for energy acquisition because it can convert food into host nutrition, and the obesity-associated gut microbiome has an increased ability to harvest energy from the diet (Turnbaugh et al., 2006). In obese individuals, there are obvious changes in microbiome composition, such as *Akkermansia*, *Faecalibacterium*, *Oscillibacter*, and *Alistipes*, which show a significant decrease. Obesity was associated with alterations in serum metabolites that were correlated with gut microbial patterns (Thingholm et al., 2019). In addition, the abundance of *Bacteroides thetaiotaomicron*, a glutamate-fermenting commensal, was significantly decreased and negatively correlated with serum glutamate concentration in obese individuals. Consistently, oral administration of *B*. *thetaiotaomicron* reduced plasma glutamate concentrations in mice, reducing diet-induced weight gain and obesity (Liu et al., 2017a).

**Table 1:** Changes in the gut microbiome in metabolic diseases.

	Up-regulated	Down-regulated	References
Obesity	Mollicutes	*Akkermansia*	Turnbaugh et al., (2008); Thingholm et al., (2019); Liu et al., (2017)
		*Faecalibacterium*	
		*Oscillibacter*	
		*Alistipes*	
		*Bacteroides thetaiotaomicron*	
NAFLD	Bacteroidetes	Firmicutes	Da Silva et al., (2018); Wang et al., (2016)
PCOS	*Prevotella*	*Lactobacillus*	Kelley et al., (2016); Guo et al., (2016); Liu et al., (2017)
	*Bacteroides*	*Ruminococcus*	
	*Streptococcus*	*Clostridium*	
		*Akkermansia*	
		*Ruminococcaceae*	

Previous studies have shown that the gut microbiome has a significant influence on the development of NAFLD in humans. Patients with NAFLD had an increased abundance of the phylum Bacteroidetes, while that of the short-chain fatty acid-producing and 7α-dehydroxylating Firmicutes was significantly decreased (Wang et al., 2016; Da Silva et al., 2018). A study using a transplantation mouse model demonstrated the role of the gut microbiota in NAFLD development. Mice fed a high-fat diet developed hepatic macrovesicular steatosis after colonization with microbiota from hyperglycemic mice, while the control mice that were treated with microbiota from normoglycemic mice developed only low-level steatosis. Differences in microbiota composition can determine the response of mice to a HFD (Le Roy et al., 2013). A prospective study demonstrated that NAFLD and NASH were associated with intestinal dysbiosis, the fecal microbiomes of children with NAFLD had lower α-diversity than those of control children, and the fecal microbiomes of children with nonalcoholic steatohepatitis had the lowest α-diversity. Moreover, the abundance of genes encoding inflammatory bacterial products was related to NAFLD and its severity. Alterations of the gut microbiome might cause the pathogenesis of NAFLD and serve as markers of disease or severity (Schwimmer et al., 2019).

PCOS is a heterogeneous endocrine disorder and the most common endocrine condition in women of reproductive age (Norman et al., 2007). Similarly, metabolic disorders have been proposed to explain the pathogenesis of PCOS. A possible relation between dysbiosis of gut microbiota and PCOS emerged from a study by Kelley et al. (Kelley et al., 2016), in which there were significant changes in the composition of the gut microbiome in a letrozole-induced mouse model of PCOS. Letrozole treatment of peripubertal female mice decreased mouse gut bacterial diversity and precipitated species-specific and time-dependent shifts in the relative abundance of particular Bacteroidetes and Firmicutes (Kelley et al., 2016). In particular, *Lactobacillus*, *Ruminococcus* and *Clostridium* were lower, while *Prevotella* was higher in letrozole-treated PCOS rats (Guo et al., 2016). In 2017, a study demonstrated that patients with PCOS have reduced diversity and an altered phylogenetic profile in their stool microbiome, which is associated with clinical parameters (Lindheim et al., 2017). Additionally, the bacteria belonging to *Bacteroides*, *Escherichia*/*Shigella* and *Streptococcus* was increased in patients with PCOS, while the bacteria from *Akkermansia* and *Ruminococcaceae* decreased (Liu et al., 2017b). According to the above mentioned studies, the gut microbiome exerts a significant influence on systemic metabolic homeostasis, and a healthy gut microbiome plays an important role in the overall health of the host.

## THE MAIN METABOLITES PRODUCED IN THE GUT MICROBIOME

The human gut microbiome is fueled by dietary macronutrients to produce bioactive compounds consisting of bile acids, short-chain fatty acids, ammonia, phenols, endotoxins and so on. These microbiota-derived metabolites are agents of microbe-host communication, which is essential for maintaining host physiology (Schroeder and Backhed, 2016). In fact, metabolite profiles associated with the gut microbiome provide further insights into the impact of lifestyle and dietary factors on diseases. Many meta-omics approaches have been developed to investigate the function of microbiota-derived metabolites, including metagenomics, metaproteomics, and metabolomics. These technologies allow us to define metabolic profiles, identify and quantify categories and compounds of interest, characterize small molecules produced by the gut microbiome and define the biochemical pathways of metabolites (Vernocchi et al., 2016). Here, we summarize the most studied findings about the significance of microbe-derived metabolites in metabolic diseases (Table 2).

**Table 2:** The role of microbe-derived metabolites in metabolic diseases.

	Metabolites	Functions	References
Bile acids	TUDCA	Hepatic and muscle insulin sensitivity↑	Kars et al., (2010)
	TβMCA	Glucose intolerance↓	Sun et al., (2019)
	GUDCA	Hyperglycemia↓	Sun et al., (2018)
	GDCA	Insulin resistance↓	Qi et al., (2019)
SCFAs	Propionate and butyrate	Energy intake↑	Larraufie et al., (2018); Psichas et al., (2015); Tolhurst et al., (2012)
	PA	Leptin↑	Al-Lahham et al., (2010)
	Acetate	Appetite and nutrition intake↓	Frost et al., (2014)
	Butyrate and FBA	Hepatic fat accumulation and insulin resistance↓	Li et al., (2018)
Other metabolites	Ethanol	Epithelial tight junctions↓	Rao et al., (2004)
	HYA	Obesity↓	Miyamoto et al., (2019)
	Ceramide	Cold-induced thermogenesis↓	Zhang et al., (2019)
	Taurine, histamine, and spermine	IL-18↑	Levy et al., (2015)
	Indole-3-aldehyde	IL-22↑	Zelante et al., (2013)

TUDCA, tauroursodeoxycholic acid; TβMCA, tauro-β-muricholic acid; GUDCA, glycoursodeoxycholic acid; GDCA, glycodeoxycholic acid; SCFAs, short-chain fatty acids; PA, propionic acid; FBA, N-(1-carbamoyl-2-phenyl-ethyl) butyramide; HYA, 10-hydroxy-cis-12-octadecenoic acid.

### Bile acids

Primary bile acids are converted from cholesterol in the liver and taurine and glycine conjugates for secretion into the gut, where they are transformed into secondary bile acids by bile salt hydrolase (BSH) in the gut microbiome (Matsubara et al., 2013). Bile acids alter metabolism by activating certain receptors, including the farnesoid X receptor (FXR), pregnane X receptor and G protein-coupled receptors (GPCRs), such as TGR5 (Matsubara et al., 2013). The secondary bile acids deoxycholic acid (DCA) and lithocholic acid (LCA) are the most abundant metabolites in the gut microbiome, accumulating at concentrations of approximately 500 μmol/L and modulating host energy homeostasis and metabolism via the G-protein-coupled receptor TGR5 (Duboc et al., 2014). Activation of intestinal FXR induces the expression of fibroblast growth factor 15 (FGF15) and inhibits the expression of cholesterol 7α-hydroxylase (CYP7A1) in the liver. CYP7A1 is a rate-limiting step for bile acid synthesis, thus leading to a decrease in bile acid levels through a gut-microbiota-liver feedback loop.

The gut microbiome is an important modulator of bile acid metabolism, and bile acid diversity is reduced in germ-free and antibiotic-treated rats. In particular, there is a major increase in the abundance of taurine-conjugated bile acids in germ-free and antibiotic-treated rats (Swann et al., 2011). Treatment with the antidiabetic medication acarbose changes the gut microbiome involved in bile acid metabolism, thereby altering bile acid composition and influencing the outcome of type 2 diabetes patients (Gu et al., 2017). A cohort study showed that treatment with tauroursodeoxycholic acid (TUDCA), a bile acid derivative, improved hepatic and muscle insulin sensitivity (Kars et al., 2010). It was reported that the important enzyme in the bile acid synthesis pathway cytochrome P450, family 7, subfamily b, polypeptide 1 (CYP7B1)-mediated activation of the alternative pathway exerted metabolic benefits by changing the gut microbiota and upregulating cold-induced thermogenesis and energy expenditure (Worthmann et al., 2017). Antibiotic treatment up-regulated CYP7B1 and increased tauro-β-muricholic acid (TβMCA) in the hamster and suppressed intestinal FXR signaling, thereby alleviating high-fat diet-induced glucose intolerance and hepatic steatosis (Sun et al., 2019). Administration of bile acid has also been shown to affect the composition of gut microbiota in turn (Islam et al., 2011). The host-microorganism biliary network also has a pivotal role in shaping host immune responses. According to a recent study from Harvard University, the intestinal bile acid pool modulates an important population of colonic RORγ^+^ regulatory T cells and affects host susceptibility to inflammatory colitis via BA nuclear receptors (Song et al., 2020). Two distinct derivatives of LCA, 3-oxoLCA and isoalloLCA, have recently been identified as T cell regulators in mice. The administration of 3-oxoLCA and isoalloLCA to mice reduced T_H_17 cell differentiation and increased Treg cell differentiation, respectively, in the intestinal lamina propria (Hang et al., 2019). With mass spectrometry informatics and data visualization approaches, a research team from the University of California San Diego found the amino acid conjugations of host bile acids that were used to produce phenylalanocholic acid, tyrosocholic acid and leucocholic acid, which were not previously characterized. These bile acid conjugates were enriched in patients with inflammatory bowel disease or cystic fibrosis and are agonists of the FXR *in vitro*. Further studies are required to confirm whether these compounds have physiological effects in the host and whether they play a role in diseases that are associated with microbiome dysbiosis (Quinn et al., 2020).

**Figure 1:**
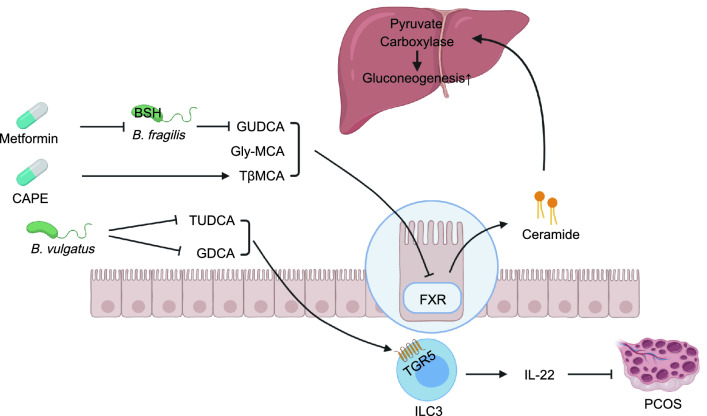
**The modulation of the gut microbiome-bile acid-FXR axis**. Treatment with metformin decreased the abundance of *Bacteroides fragilis* and increased the level of GUDCA, thereby suppressing intestinal FXR signaling. Gly-MCA inhibited intestinal FXR signaling and altered host liver lipid metabolism. CAPE supplementation inhibited bacterial BSH to increase the levels of intestinal TβMCA, which selectively suppressed intestinal FXR signaling and reduced the level of ceramide, thereby reducing hepatic gluconeogenesis in mice. *Bacteroides vulgatus* can deconjugate GDCA and TUDCA synthesized in individuals with PCOS. GDCA can induce ILC3 secretion of IL-22 through TGR5, GATA binding protein 3, and IL-22, in turn improving the PCOS phenotype. CAPE, caffeic acid phenethyl ester; GUDCA, glycoursodeoxycholic acid; Gly-MCA, glycine-β-muricholic acid; TβMCA, tauro-β-muricholic acid; TUDCA, tauroursodeoxycholic acid; GDCA, glycodeoxycholic acid; ILC3, intestinal group 3 innate lymphoid cell; PCOS, polycystic ovary syndrome

Modulation of the gut microbiome-bile acid-FXR axis is associated with obesity-induced insulin resistance and hepatic steatosis in mice. Previous relevant work in our laboratory is summarized in Fig 1. Our team has revealed that ablation of the gut microbiome alleviates HFD-induced glucose intolerance, hepatic steatosis and inflammation by modulating the key enzyme CYP7A1 in the alternative bile acid synthesis pathway in hamsters, thus providing a potential target to modulate diet-induced obesity (Sun et al., 2019). We identified glycine-β-muricholic acid (Gly-MCA) as a selective high-affinity inhibitor of intestinal FXR signaling that can reverse HFD-induced obesity by reducing the biosynthesis of intestinal-derived ceramides, which directly compromises beige fat thermogenic function (Jiang et al., 2015). We further showed that Gly-MCA inhibited intestinal FXR as an antagonist, thus altering host liver lipid metabolism and improving obesity-related metabolic dysfunction (Zhang et al., 2016). These data suggest that Gly-MCA may be a candidate for the treatment of metabolic disorders. In addition, we found that supplementation with an inhibitor of bacterial BSH, caffeic acid phenethyl ester (CAPE), increased the levels of intestinal TβMCA, which selectively suppressed intestinal FXR signaling and decreased ceramide levels, thus attenuating hepatic gluconeogenesis in mice. These results suggested that inhibiting intestinal FXR is a strategy for treating hyperglycemia (Xie et al., 2017). Our study was further verified by suppression of intestinal FXR signaling using theabrownin, which is a key component of Pu-erh tea. Oral administration of theabrownin suppressed the activity of intestinal bacterial BSH activity, resulting in the accumulation of conjugated bile acids, which inhibited intestinal FXR-FGF15 signaling, thus ultimately reducing the level of cholesterol (Huang et al., 2019). Our team has reported that metformin treatment decreased the abundance of *Bacteroides fragilis* and increased the level of glycoursodeoxycholic acid (GUDCA) in the gut, thereby suppressing intestinal FXR signaling. The antihyperglycemic effect of metformin occurs mainly through a *B. fragilis*-GUDCA-intestinal FXR axis (Sun et al., 2018).

In addition to interfering with the production of ceramides, bile acids can also particularly act on the FXR in L-cells and control glucagon-like peptide-1 (GLP-1) production. Treatment with the bile acid sequestrant colesevelam increases GLP-1 secretion and improves glycemia in *ob*/*ob* mice in an FXR-dependent manner, thus confirming that the FXR/GLP-1 pathway is a new mechanism for bile acids to control glucose metabolism and a pharmacological target for type 2 diabetes (Trabelsi et al., 2015). It has also been shown that bile diversion to the ileum (GB-IL) has similar metabolic benefits to Roux-en-Y gastric bypass (RYGB) via the bile acid-intestinal FXR-GLP-1 axis (Albaugh et al., 2019). In addition, previous research in our laboratory has reported that *Bacteroides vulgatus* was markedly elevated in the gut microbiota of individuals with PCOS, which deconjugated conjugated bile acids, such as glycodeoxycholic acid (GDCA) and TUDCA. GDCA induced intestinal group 3 innate lymphoid cell IL-22 secretion through TGR5, GATA binding protein 3, and IL-22, in turn improving the PCOS phenotype. Transplantation of fecal microbiota from women with PCOS or *B*. *vulgatus*-colonized recipient mice resulted in increased disruption of ovarian function, insulin resistance, altered bile acid metabolism, and reduced interleukin-22 secretion and infertility (Qi et al., 2019). Therefore, targeting the gut microbiome to alter intestinal bile acid composition or directly targeting the bile acid receptors FXR and TGR5 may be promising potential therapeutic interventions for metabolic diseases.

### Manipulation to modulate bile acids

It is well established that the gut microbiome is crucial for maintaining the physiological state and metabolic homeostasis of the host. The gut microbiome of patients with metabolic diseases is maladjusted, and the interaction between the gut microbiome and host is disordered. Therefore, regulation of the host gut microbiome may be a prospective method for the treatment of metabolic diseases. Gut microbiome colonization is thought to begin primarily at birth when the infant is exposed to the maternal microbiota during delivery (Perez-Munoz et al., 2017). A variety of factors in early life can influence the composition of the gut microbiome, including the mode of delivery, host genetics, immune responses, antibiotic administration, lifestyle, circadian rhythm, host disease state and environment (Gilbert et al., 2018). A cohort study demonstrated that the gut microbiome is shaped predominantly by environmental factors and that host genetics have a minor role in determining microbiome composition. In addition, other lifestyle traits have been shown to correlate with the composition of the microbiota, including sleep deprivation, stress, occupation, and sexual intercourse in heterosexual couples (Gilbert et al., 2018). Knowing the factors that affect the gut microbiome, we can use corresponding methods to regulate the gut microbiome to manipulate the bile acid pool of the body to improve metabolic diseases.

#### Effect of dietary intervention on the composition of the gut microbiome and bile acids

Throughout the lifetime, diet may have the greatest impact on the relationship between the gut microbiome and its mammalian host. The consumption of various nutrients affects the structure of the microbial community and provides a substrate for microbial metabolism (Albenberg and Wu, 2014). The gut microbiome interacts with nutrients in the diet to influence the health of the host. Furthermore, the structure and activity of the gut microbiome is largely modulated by dietary intake in humans, and the process is rapid and repeatable. Thus, diet intervention is a powerful tool for changing the composition of the gut microbiome. There were significant differences in the composition of the gut microbiome between herbivorous and carnivorous individuals, and the carnivorous diet increased the abundance of bile-tolerant microorganisms and decreased the levels of Firmicutes that metabolize dietary plant polysaccharides such as *Roseburia*, *Eubacterium rectale* and *Ruminococcus bromii* (David et al., 2014). Omnivorous human subjects who have high intake of fat and protein have increased levels of *Bacteroides*, whereas vegetarians who have high fiber intake have increased levels of *Prevotella.* Barley kernel-based meal, which is rich in non-starch polysaccharides and resistant starch, improves glucose metabolism in healthy subjects whose *Prevotella*/*Bacteroides* ratio was higher, causing *Prevotella copri* to have increased potential to ferment complex polysaccharides (Kovatcheva-Datchary et al., 2015). Fecal communities aggregated into enterotypes and were mainly distinguished by the levels of Bacteroides and Prevotella. Intestinal types are closely related to long-term diet, particularly proteins and animal fats (Bacteroidetes) versus carbohydrates (Prevotella). Changes in the composition of the microbiome were detected within 24 hours of the initiation of high-fat/low-fiber or low-fat/high-fiber diets, but the characteristics of enterotype remained stable over the 10-day study period (Wu et al., 2011). Therefore, the corresponding dietary intervention needs to not only control the nutrients in the diet but also pay attention to the time of dietary intervention to ensure sufficient time to change the targeted gut microbiome. A parallel 8-week randomized controlled trial demonstrated that a Mediterranean diet intervention in overweight and obese subjects lowered plasma cholesterol and caused changes in the gut microbiome and fecal bile acids, significantly reducing fecal concentrations of total bile acids, including both primary and secondary bile acids. In particular, fecal DCA and LCA were significantly reduced after 4 and 8 weeks of the intervention (Meslier et al., 2020). In contrast, a HFD markedly increases bile acid levels in the intestinal lumen and serum and alters bile acid profiles, including disproportionate increases in the levels of primary bile acids and secondary bile acids. Of particular interest, the levels of TβMCA, βMCA, DCA and ωMCA were significantly increased with HFD feeding (Fu et al., 2019). A high-protein diet causes a greater abundance of the bacteria Eubacterium, which is capable of bile acid 7α-dehydroxylation, also resulting in higher levels of secondary bile acids DCA and LCA (Pi et al., 2020). Methionine restriction attenuated insulin resistance triggered by HFD and restored the periodic fluctuation of lipidolysis genes and bile acid synthetic genes interrupted by HFD, thus improving the circulating lipid profile (Wang et al., 2020). A recent study revealed that new food ingredient-extruded legumes plus cereal mixes can modulate lipid profiles and increase fecal excretion of bile acids (Rubio et al., 2020). This research drives our attention to the nutritional and physiological effects of extruded legumes plus cereal mixes. In addition, common buckwheat (Fagopyrum esculentum Moench.) protects against HFD-induced NAFLD associated with dyslipidemia in mice. Common buckwheat supplementation had significant regulatory effects on primary bile acid biosynthesis and altered the structure of the gut microbiome, thus improving lipid metabolism. This study demThroughout the lifetime, diet may have the greatest impact on the relationship between the gut microbiome and its mammalian host. The consumption of various nutrients affects the structure of the microbial community and provides a substrate for microbial metabolism (Albenberg and Wu, 2014). The gut microbiome interacts with nutrients in the diet to influence the health of the host. Furthermore, the structure and activity of the gut microbiome is largely modulated by dietary intake in humans, and the process is rapid and repeatable. Thus, diet intervention is a powerful tool for changing the composition of the gut microbiome. There were significant differences in the composition of the gut microbiome between herbivorous and carnivorous individuals, and the carnivorous diet increased the abundance of bile-tolerant microorganisms and decreased the levels of Firmicutes that metabolize dietary plant polysaccharides such as *Roseburia*, *Eubacterium rectale* and *Ruminococcus bromii* (David et al., 2014). Omnivorous human subjects who have high intake of fat and protein have increased levels of *Bacteroides*, whereas vegetarians who have high fiber intake have increased levels of *Prevotella.* Barley kernel-based meal, which is rich in non-starch polysaccharides and resistant starch, improves glucose metabolism in healthy subjects whose *Prevotella*/*Bacteroides* ratio was higher, causing *Prevotella copri* to have increased potential to ferment complex polysaccharides (Kovatcheva-Datchary et al., 2015). Fecal communities aggregated into enterotypes and were mainly distinguished by the levels of Bacteroides and Prevotella. Intestinal types are closely related to long-term diet, particularly proteins and animal fats (Bacteroidetes) versus carbohydrates (Prevotella). Changes in the composition of the microbiome were detected within 24 hours of the initiation of high-fat/low-fiber or low-fat/high-fiber diets, but the characteristics of enterotype remained stable over the 10-day study period (Wu et al., 2011). Therefore, the corresponding dietary intervention needs to not only control the nutrients in the diet but also pay attention to the time of dietary intervention to ensure sufficient time to change the targeted gut microbiome. A parallel 8-week randomized controlled trial demonstrated that a Mediterranean diet intervention in overweight and obese subjects lowered plasma cholesterol and caused changes in the gut microbiome and fecal bile acids, significantly reducing fecal concentrations of total bile acids, including both primary and secondary bile acids. In particular, fecal DCA and LCA were significantly reduced after 4 and 8 weeks of the intervention (Meslier et al., 2020). In contrast, a HFD markedly increases bile acid levels in the intestinal lumen and serum and alters bile acid profiles, including disproportionate increases in the levels of primary bile acids and secondary bile acids. Of particular interest, the levels of TβMCA, βMCA, DCA and ωMCA were significantly increased with HFD feeding (Fu et al., 2019). A high-protein diet causes a greater abundance of the bacteria Eubacterium, which is capable of bile acid 7α-dehydroxylation, also resulting in higher levels of secondary bile acids DCA and LCA (Pi et al., 2020). Methionine restriction attenuated insulin resistance triggered by HFD and restored the periodic fluctuation of lipidolysis genes and bile acid synthetic genes interrupted by HFD, thus improving the circulating lipid profile (Wang et al., 2020). A recent study revealed that new food ingredient-extruded legumes plus cereal mixes can modulate lipid profiles and increase fecal excretion of bile acids (Rubio et al., 2020). This research drives our attention to the nutritional and physiological effects of extruded legumes plus cereal mixes. In addition, common buckwheat (Fagopyrum esculentum Moench.) protects against HFD-induced NAFLD associated with dyslipidemia in mice. Common buckwheat supplementation had significant regulatory effects on primary bile acid biosynthesis and altered the structure of the gut microbiome, thus improving lipid metabolism. This study demonstrated that common buckwheat can be used as a potential functional food for the prevention of NAFLD and hyperlipidemia (Huang et al., 2020). Recently, intermittent fasting, which includes time-restricted feeding (TRF: limiting daily food consumption to a 4- to 12-hour window without limiting caloric intake) and every-other-day fasting (EODF), has been demonstrated to optimize energy metabolism and is considered a potent diet and lifestyle intervention that ameliorates and postpones the onset of metabolic diseases (di Francesco et al., 2018). Preclinical studies and clinical trials have shown that TRF can confer protection against many health problems, such as obesity (Hatori et al., 2012), diabetes mellitus (Eizabeth et al., 2018) and cardiovascular disease (Brandhorst and Longo, 2019). However, the molecular mechanism responsible for the benefits of TRF is not entirely clear. Until now, it has been shown to be related to the circadian rhythm (Longo and Panda, 2016), the ketogenic pathway (da Cabo and Mattson, 2019) and the gut microbiome. EODF treatment results in changes in gut microbiome composition, significantly increasing the abundance of Firmicutes while decreasing Bacteroidetes and Tenericutes. The shift in the gut microbiome composition results in an increase in acetic acid and lactic acid as fermentation products and selectively stimulates beige fat development within white adipose tissue, dramatically ameliorating obesity, insulin resistance and hepatic steatosis (Li et al., 2017). According to the above experimental results, dietary interventions are potential tools for modulating the gut microbiome and further impacting host health.

#### Gut-targeted drug for metabolic diseases

In addition to dietary interventions, drugs are the main intervention strategies for metabolic diseases. The gut microbiome is widely recognized as a major regulator of host health and drugs that drive changes in microbial composition and function with important consequences for host health. The gut microbiome interacts with a variety of common antidiabetic drugs, including metformin, thiazolidinedione, miglitol, acarbose, and liraglutide (Whang et al, 2019). For example, metformin, the first-line drug for the treatment of T2DM, has recently been identified as a key contributor to changes in the human gut microbiome composition in patients with T2DM (Forslund et al., 2015). Mechanistically, we found that metformin treatment increased the level of the bile acid GUDCA by inhibiting *B. fragilis* and BSH activity, thus improving insulin resistance through an intestinal FXR-dependent pathway, which raised the treatment potential of the *B. fragilis*-GUDCA-intestinal FXR axis (Sun et al., 2018). Metformin treatment increased the abundance of *Lactobacillus* in the upper small intestine and restored the expression of sodium glucose cotransporter-1 (SGLT1), thus increasing glucose sensitivity (Bauer et al., 2018). A double-blind study demonstrated that metformin treatment resulted in a significant shift in the relative abundance of many bacterial strains, including an increase in *Escherichia* and a decrease in *Intestinibacter.* The results of fecal bacteria transplantation showed that metformin-treated donors’ gut microbiome can improve glucose tolerance. Moreover, metformin treatment increased the expression of BSH in the gut microbiome, resulting in an increase in the concentration of unconjugated bile acids in plasma (Wu et al., 2017). Berberine is also therapeutic for T2DM and its associated complications, such as peripheral neuropathy, kidney disease, and cardiomyopathy (Zhang et al., 2015). Intervention with berberine increased the SCFA-producing bacteria (e.g., *Butyricimonas*, *Coprococcus*, and *Ruminococcus*) and other probiotics, including *Lactobacillus* and *Akkermansia*. SCFAs play important roles in inflammation suppression, thus promoting the integrity of the intestinal epithelium (Zhang et al., 2019). On one hand, several orally administered drugs are processed by intestinal microbial enzymes before they are absorbed into the bloodstream, so the metabolic capacity of the gut microbiome can affect the absorption and function of these drugs (Kim, 2015). Conversely, drugs can manipulate the composition and function of the gut microbiome composition. Therefore, understanding the bidirectional interactions between drugs and the gut microbiome and their impact on the clinical outcome of treatment for metabolic diseases may provide insights for the development of strategies to improve metabolic diseases in the next generation.

#### Probiotic supplements

Currently, probiotic therapy is commonly used to prevent metabolic diseases, such as diabetes (Sun and Buys, 2016) and NAFLD (Aron-Wisnewsky et al., 2020). To study the effects of probiotics on the host, assessing whether probiotics colonize the gut is crucial. A recent systematic review reported that six of the seven analyzed studies found no effect of probiotics on fecal microbiome composition (Kristensen et al., 2016), as reported in other studies (Eloe-Fadrosh et al., 2015; Laursen et al., 2017). In contrast, other studies have observed changes in the fecal microbiome composition of probiotic-treated individuals (Ferrario et al., 2014; Wang et al., 2015). Because probiotics can hardly colonize the recipients’ gut, prebiotics, which were defined as “a substrate that is selectively utilized by host microorganisms conferring a health benefit (Gibson et al., 2017)”, are necessary to manipulate the gut microbiome by promoting the propagation of probiotics. In addition, there is an urgent need to develop a way to efficiently deliver probiotics to the intestine where microbiota naturally resides. A recent article published by researchers at Wake Forest University designed a novel system for the effective targeted delivery of ingested probiotics. They encapsulated the bacterial cells in the hydrogel of the modified alginate, thus protecting the bacteria from destruction by the acidic stomach, making it effective for orally delivery of probiotics (Enck et al., 2020). Recent research has suggested that *Lactobacillus casei* YRL577 can alleviate NAFLD by modifying genes in the intestinal bile acid pathway (Zhang et al., 2020). Furthermore, *Lactobacillus plantarum* TAR4 supplementation *in vivo* reduced the absorption of bile acids for enterohepatic recycling and increased the catabolism of cholesterol to bile acids, providing a new nonpharmacological alternative to reduce cardiovascular risk factors (Lim et al., 2020). The use of probiotics and how they impact the gut microbiome and bile acids is a growing and promising field for the treatment of a variety of metabolic diseases.

#### Genetic manipulation of the gut microbiome

In addition to the dietary intervention and probiotics mentioned above, there are other practices that can regulate the composition of the gut microbiome. A recent article published by investigators at Stanford University has developed a system for constructing clean deletions in *Clostridium* spp. to determine the function of microbial products. *Clostridium* is a symbiotic bacterium of the Firmicutes phylum that is commonly found in the gut of mammals. *Clostridia* produce a series of metabolites that spread into the circulation of its host and are genetically difficult to manipulate. To study the effects of molecules produced by the gut microbiome, Guo et al. developed a genetic system based on CRISPR-Cas9 for constructing deletions in a model commensal *Clostridium*, *Clostridium sporogenes*, stopping the production of specific molecules (Guo et al., 2019). Germ-free mice colonized with mutants exhibited altered production of immunoglobulin A, which is involved in barrier protection in mucous membranes. This finding suggests that we can change the metabolites produced by the gut microbiome through genetic manipulation to study the interactions between metabolites and hosts. Nonetheless, despite the obvious impact of bile acids on host physiology, our ability to regulate secondary bile acid levels in the host is limited by incomplete understanding of their biosynthetic genes and a lack of genetic tools. A recent article published by a group at Stanford University established a system for engineering the key enzymes in the conversion of cholic acid to DCA in *Clostridium sporogenes*, conferring the ability to produce DCA and LCA to a nonproducing commensal and demonstrating that a microbiome-derived pathway can be expressed and controlled heterologously (Funabashi et al., 2020). Genetic abolition of bile acid metabolic pathways in Bacteroides substantially decreased the colonic RORγ^+^ regulatory T cell population, thus aggravating host susceptibility to inflammatory colitis (Song et al., 2020).

### Other metabolites

Substances in food have a significant role in shaping the composition of the gut microbiome. The majority of colonic microbiota add available proteins and amino acids to their own biomass and enzymatic mechanisms, in which case they prefer indigestible fermentable carbohydrates in foods, including poorly absorbed carbohydrates, resistant starch, and probiotics, thus generating important metabolites such as short-chain fatty acids (SCFAs) and succinate. For the microbiome colonized in the distal colon, fermentable carbohydrates are depleted, and microorganisms turn to protein fermentation, mainly producing detrimental metabolites such as ammonia, phenols and branched-chain fatty acids (Canfora et al., 2019).

SCFAs, particularly acetate, propionate, and butyrate, are the main products of microbial fermentation activity in the gut (Cummings et al., 1987). For example, acetate can be produced from pyruvate by many gut bacteria, such as *B. hydrogenotrophica*, via acetyl-CoA or the Wood-Ljungdahl pathway (Ragsdale and Pierce, 2008). Most of the propionate is formed via the succinate pathway by Bacteroidetes and by some Firmicutes that belong to the Negativicutes class (such as *Phascolarctobacterium succinatutens*, *Dialister* spp. and *Veillonella* spp.) (Louis et al., 2014). SCFAs, such as propionate and butyrate, have the ability to affect energy intake and insulin secretion by producing satiety hormone. SCFAs trigger secretion of glucagon-like peptide (GLP)-1 and peptide YY (PYY) in enteroendocrine cells through the GPCRs GPR43 (FFAR2) and GPR41 (FFAR3) (Tolhurst et al., 2012; Psichas et al., 2015; Larraufie et al., 2018). In addition, propionic acid (PA) can stimulate the expression of the anorexigenic hormone leptin in human adipose tissue (Al-Lahham et al., 2010). These results suggest that SCFAs and their receptors are potential targets for the treatment of obesity and type 2 diabetes. In addition to directly affecting hormone production, SCFAs can also suppress appetite through the central nervous system. Colonic acetate can cross the blood-brain barrier and reach the hypothalamus, where acetate increases the glutamate-glutamine and GABA neuroglial cycles, increasing the production of lactate, which suppresses appetite and nutrition intake (Frost et al., 2014). Consistently, oral butyrate administration prevented diet-induced obesity, hepatic steatosis and insulin resistance by reducing food intake, which was mainly accompanied by suppression of activity of orexigenic neurons that express neuropeptide Y in the hypothalamus and reduction in neuronal activity within the nucleus tractus solitarius and dorsal vagal complex in the brainstem (Li et al., 2018). A microbiome-wide association study based on 952 normoglycemic individuals found that increased intestinal production of the SCFA butyrate was associated with improved insulin response after an oral glucose tolerance test. However, abnormalities in the production or absorption of another SCFA, propionate, were causally related to an increased risk of T2D (Sanna et al., 2019).

In addition to the metabolites mentioned above, there are many other metabolites of the gut microbiome that play a nonnegligible role in metabolic diseases, which will be only briefly introduced here. NASH, a serious liver disease associated with obesity, is characterized by metabolic syndrome, liver steatosis and liver inflammation and is thought to be influenced by the gut microbiome. Ethanol is a microbial metabolite derived from saccharolytic fermentation and cross-breeding of microorganisms. NASH and obese patients have an increased abundance of alcohol-producing bacteria consisting of *Proteobacteria* and *Enterobacteriaceae* in their feces, as well as an increased concentration of ethanol in the systemic circulation (Zhu et al., 2013). Gut bacteria-derived ethanol is possibly involved in the pathogenesis of alcoholic liver disease by disrupting epithelial tight junctions and increasing endotoxin-mediated hepatocellular damage (Rao et al., 2004). In addition to ethanol, protein fermentation metabolites may be involved in NAFLD progression. High-protein and reduced-carbohydrate diets alter the colonic microbiome, increasing ammonia, phenol and hydrogen sulfide concentrations, which cause mucosal inflammation and compromise gut epithelial structure and gut permeability (Yao et al., 2016). In addition, a study in 2019 reported that the gut microbiota confers host resistance to HFD-induced obesity by modulating dietary polyunsaturated fatty acid (PUFA) metabolism by producing the gut microbial metabolite 10-hydroxy-cis-12-octadecenoic acid (HYA) (Miyamoto et al., 2019). Research in our laboratory reported that cold exposure upregulated adipocyte hypoxia-inducible factor 2α (HIF-2α), targeting the Acer2 gene, which encodes alkaline ceramidase 2, triggering ceramide catabolism and mediating cold-induced thermogenesis, thereby alleviating atherosclerosis (Zhang et al., 2019).

Immunity also plays an important role in metabolic diseases. The gut microbiome has a highly coevolutionary relationship with the host immune system, and many of these microbes are essential to host physiology. Emerging evidence suggests that the mammalian immune system plays a crucial role in maintaining the homeostasis of the resident microbiome (Bain and Cerovic, 2020). SCFAs are inhibitors of histone deacetylases (HDACs) and ligands for GPCRs and thereby act as signaling molecules that influence the immune system. SCFAs (butyrate, propionate, and acetate) affect peripheral blood mononuclear cells as HDAC inhibitors, resulting in inactivation of nuclear factor-κB (NF-κB) and lower production of the proinflammatory cytokine tumor necrosis factor-α (TNF-α) (Usami et al., 2008). An additional study further demonstrated that the SCFA n-butyrate can modulate the function of intestinal macrophages through inhibition of histone deacetylases to downregulate the production of proinflammatory mediators, including nitric oxide, IL-6, and IL-12 (Chang et al., 2014). Inflammasome signaling is involved in regulating the integrated intestinal host-commensal microenvironment, and the microbial metabolites taurine, histamine, and spermine modulate the host-microbiome interface by co-modulating NLRP6 inflammasome signaling, epithelial IL-18 secretion, and downstream antimicrobial peptide (AMP) profiles (Levy et al., 2015). Lipopolysaccharides (LPSs) can cause metabolic endotoxemia characterized by low levels of inflammation, insulin resistance, and increased cardiovascular risk. Moreover, LPSs can trigger the release of proinflammatory molecules, which interfere with the modulation of glucose and insulin metabolism, promote the development and rupture of atherosclerotic plaques and are conducive to the development of fatty liver disease (Manco et al., 2010). The aryl hydrocarbon receptor (AHR) is a ligand-inducible transcription factor that is expressed by immune cells and epithelial cells, where metabolites of the gut microbiome can bind the AHR to regulate mucosal immune responses. *Lactobacilli spp.* can metabolize dietary tryptophan and generate the AHR ligand indole-3-aldehyde, which can stimulate group 3 innate lymphoid cells (ILC3s), thereby inducing IL-22 production. IL-22 upregulates the expression of AMP, which provide colonization resistance to pathogens such as the fungus *Candida albicans* (Zelante et al., 2013). A recent study showed that ILC3s persistently produce IL-22 in the absence of adaptive CD4^+^ T cell activity, leading to impaired host lipid metabolism by reducing the expression of lipid transporters in the small intestine (Mao et al., 2018). These findings provide new insights into how metabolites of the gut microbiome affect tissue metabolic homeostasis; thus, manipulation of the diet and gut microbiome may have potential therapeutic applications in the prevention and treatment of metabolic disorders.

## CONCLUSIONS AND FUTURE PERSPECTIVES

We are living with an enormous number of microorganisms in our guts, ranging from bacteria, viruses, fungi, and archaea to phages and protozoa. The gut microbiome can modulate nutrient metabolism upon dietary intake and produce many metabolites to interact with the host in a variety of ways, including regulating glucose and lipid metabolism pathways, influencing the differentiation and function of immune cells, affecting insulin sensitivity and so on. An overwhelming amount of human and animal data provides strong evidence of a crucial role of the gut microbiome and its metabolites in the occurrence and development of many metabolic diseases.

Based on the results of recent research and experiments, we have found many ways to improve metabolic diseases by regulating the gut microbiome, including dietary intervention, administration of probiotics, gene editing technology and drug use. Apart from the abovementioned application, we can also predict a person’s susceptibility to disease or response to drugs by detecting his or her microbiome features. Based on many clinical follow-up studies from different countries, most individuals (perhaps up to 70% (Shaw et al., 1999; Larsson et al., 2000; de Vegt et al., 2001; Tuomilehto et al., 2001; Knowler et al., 2002; Vendrame and Gottlieb, 2004; Santaguida et al., 2005)) with prediabetic states, including impaired fasting glucose (IFG) and impaired glucose tolerance (IGT), may eventually develop type 2 diabetes (Nathan et al., 2007). In addition, prediabetes is closely associated with other manifestations, including obesity, hypertension, nonalcoholic fatty liver disease, hypertriglyceridemia, and cardiovascular disease (Grundy, 2012). A cohort study in 2015 continuously monitored week-long glucose levels in 800 subjects and collected data about their microbiomes, genetics, dietary habits, anthropometrics and physical activity. The researchers demonstrated that people respond differently to the same meal and devised a machine-learning algorithm that used personal and microbiome features to enable accurate prediction of glucose response (Zeevi et al., 2015).

There is no doubt that we have progressed considerably in the analysis of the composition and key metabolites of the gut microbiome. However, we must acknowledge that we still need to perform more work in addition to finding simple associations. The complex mechanisms underlying the interactions between the gut microbiome and host need to be further studied. In addition, new technologies should be applied to investigate and manipulate the microbiome to precisely intervene in a specific microbiome.

## References

[CR1] Albaugh VL, Banan B, Antoun J, Xiong Y, Guo Y, Ping J, Alikhan M, Clements BA, Abumrad NN, Flynn CR (2019). Role of bile acids and GLP-1 in mediating the metabolic improvements of bariatric surgery. Gastroenterology.

[CR2] Albenberg LG, Wu GD (2014). Diet and the intestinal microbiome: associations, functions, and implications for health and disease. Gastroenterology.

[CR3] Al-Lahham SH, Roelofsen H, Priebe M, Weening D, Dijkstra M, Hoek A, Rezaee F, Venema K, Vonk RJ (2010). Regulation of adipokine production in human adipose tissue by propionic acid. Eur J Clin Invest.

[CR4] Aron-Wisnewsky J, Warmbrunn M, Nieuwdorp M, Clement K (2020). Nonalcoholic fatty liver disease: modulating gut microbiota to improve severity?. Gastroenterology.

[CR5] Bain CC, Cerovic V (2020). Interactions of the microbiota with the mucosal immune system. Immunology.

[CR6] Bauere PV, Duca FA, Waise TMZ, Rasmussen BA, Abraham MA, Dranse HJ, Puri A, O’Brien CA, Lam TKT (2018). Metformin alters upper small intestinal microbiota that impact a glucose-SGLT1-sensing glucoregulatory pathway. Cell Metab.

[CR7] Brandhorst S, Longo VD (2019). Dietary restrictions and nutrition in the prevention and treatment of cardiovascular disease. Circ Res.

[CR8] Canfora EE, Meex RCR, Venema K, Blaak EE (2019). Gut microbial metabolites in obesity, NAFLD and T2DM. Nat Rev Endocrinol.

[CR9] Chang PV, Hao L, Offermanns S, Medzhitov R (2014). The microbial metabolite butyrate regulates intestinal macrophage function via histone deacetylase inhibition. Proc Natl Acad Sci USA.

[CR10] Corpeleijn E, Saris WH, Blaak EE (2009). Metabolic flexibility in the development of insulin resistance and type 2 diabetes: effects of lifestyle. Obes Rev.

[CR11] Cummings JH, Pomare EW, Branch WJ, Naylor CP, Macfarlane GT (1987). Short chain fatty acids in human large intestine, portal, hepatic and venous blood. Gut.

[CR12] da Cabo R, Mattson MP (2019). Effects of intermittent fasting on health, aging, and disease. N Engl J Med.

[CR13] Da Silva HE, Teterina A, Comelli EM, Taibi A, Arendt BM, Fischer SE, Lou W, Allard JP (2018). Nonalcoholic fatty liver disease is associated with dysbiosis independent of body mass index and insulin resistance. Sci Rep.

[CR14] David LA, Maurice CF, Carmody RN, Gootenberg DB, Button JE, Wolfe BE, Ling AV, Devlin AS, Varma Y, Fischbach MA (2014). Diet rapidly and reproducibly alters the human gut microbiome. Nature.

[CR15] de Vegt F, Dekker JM, Jager A, Hienkens E, Kostense PJ, Stehouwer CD, Nijpels G, Bouter LM, Heine RJ (2001). Relation of impaired fasting and postload glucose with incident type 2 diabetes in a Dutch population: the Hoorn Study. JAMA.

[CR16] Di Francesco A, Di Germanio C, Bernier M, de Cabo R (2018). A time to fast. Science.

[CR17] Duboc H, Taché Y, Hofmann AF (2014). The bile acid TGR5 membrane receptor: from basic research to clinical application. Dig Liver Dis.

[CR18] Eloe-Fadrosh EA, Brady A, Crabtree J, Drabek EF, Ma B, Mahurkar A, Ravel J, Haverkamp M, Fiorino AM, Botelho C (2015). Functional dynamics of the gut microbiome in elderly people during probiotic consumption. mBio.

[CR19] Enck K, Banks S, Yadav H, Welker ME, Opara EC (2020). Development of a novel oral delivery vehicle for probiotics. Curr Pharm Des.

[CR20] Ferrario C, Taverniti V, Milani C, Fiore W, Laureati M, De Noni I, Stuknyte M, Chouaia B, Riso P, Guglielmetti S (2014). Modulation of fecal clostridiales bacteria and butyrate by probiotic intervention with Lactobacillus paracasei DG varies among healthy adults. J Nutr.

[CR21] Forslund K, Hidebrand F, Nielsen T, Falony G, Le Chatelier E, Sunagawa S, Prifti E, Vieira-Silva S, Gudmundsdottri V, Pedersen KH (2015). Disentangling type 2 diabetes and metformin treatment signatures in the human gut microbiota. Nature.

[CR22] Frost G, Sleeth ML, Sahuri-Arisoylu M, Lizarbe B, Cerdan S, Brody L, Anastasovska J, Ghourab S, Hankir M, Zhang S (2014). The short-chain fatty acid acetate reduces appetite via a central homeostatic mechanism. Nat Commun.

[CR23] Fu T, Coulter S, Yoshihara E, Oh TG, Fang S, Cayabyab F, Zhu QY, Zhang T, Lelanc M, Liu SH (2019). FXR regulates intestinal cancer stem cell proliferation. Cell.

[CR24] Funabashi M, Grove TL, Wang M, Varma Y, McFadden ME, Brown LC, Guo C, Higginbottom S, Almo SC, Fischbach MA (2020). A metabolic pathway for bile acid dehydroxylation by the gut microbiome. Nature.

[CR25] Gibson GR, Hutkins R, Sanders ME, Prescott SL, Reimer RA, Salminen SJ, Scott K, Stanton C, Swanson KS, Cani PD (2017). Expert consensus document: The International Scientific Association for Probiotics and Prebiotics (ISAPP) consensus statement on the definition and scope of prebiotics. Nat Rev.

[CR26] Gilbert JA, Blaser MJ, Caporaso JG, Jansson JK, Lynch SV, Knight R (2018). Current understanding of the human microbiome. Nat Med.

[CR27] Grundy SM (2012). Pre-diabetes, metabolic syndrome, and cardiovascular risk. J Am Coll Cardiol.

[CR28] Gu Y, Wang X, Li J, Zhang Y, Zhong H, Liu R, Zhang D, Feng Q, Xie X, Hong J (2017). Analyses of gut microbiota and plasma bile acids enable stratification of patients for antidiabetic treatment. Nat Commun.

[CR29] Guo Y, Qi Y, Yang X, Zhao L, Wen S, Liu Y, Tang L (2016). Association between polycystic ovary syndrome and gut microbiota. PLoS ONE.

[CR30] Guo CJ, Allen BM, Hiam KJ, Dodd D, Van Treuren W, Higginbottom S, Nagashima K, Fischer CR, Sonnenburg JL, Spitzer MH (2019). Depletion of microbiome-derived molecules in the host using Clostridium genetics. Science.

[CR31] Hang S, Paik D, Yao L, Kim E, Trinath J, Lu J, Ha S, Nelson BN, Kelly SP, Wu L (2019). Bile acid metabolites control T(H)17 and T(reg) cell differentiation. Nature.

[CR32] Hatori M, Vollmers C, Zarrinpar A, DiTacchio L, Bushong EA, Gill S, Leblanc M, Chaix A, Joens M, Fitzpatrick JAJ (2012). Time-restricted feeding without reducing caloric intake prevents metabolic diseases in mice fed a high-fat diet. Cell Metab.

[CR33] Heymsfield SB, Wadden TA (2017). Mechanisms, pathophysiology, and management of obesity. Engl J Med.

[CR34] Huang FJ, Zheng XJ, Ma XH, Jiang RQ, Zhou WY, Zhou SP, Zhang YJ, Lei S, Wang SL, Kuang JL (2019). Theabrownin from Pu-erh tea attenuates hypercholesterolemia via modulation of gut microbiota and bile acid metabolism. Nat Commun.

[CR35] Huang ZR, Deng JC, Li QY, Cao YJ, Lin YC, Bai WD, Liu B, Rao PF, Ni L, Lv XC (2020). Protective Mechanism of Common Buckwheat (Fagopyrum esculentum Moench.) against nonalcoholic fatty liver disease associated with dyslipidemia in mice fed a high-fat and high-cholesterol diet. J Agric Food Chem.

[CR36] Islam KB, Fukiya S, Hagio M, Fujii N, Ishizuka S, Ooka T, Ogura Y, Hayashi T, Yokota A (2011). Bile acid is a host factor that regulates the composition of the cecal microbiota in rats. Gastroenterology.

[CR37] Jandhyala SM, Talukdar R, Subramanyam C, Vuyyuru H, Sasikala M, Nageshwar Reddy D (2015). Role of the normal gut microbiota. World J Gastroenterol.

[CR38] Jiang C, Xie C, Lv Y, Li J, Krausz KW, Shi J, Brocker CN, Desai D, Amin SG, Bisson WH (2015). Intestine-selective farnesoid X receptor inhibition improves obesity-related metabolic dysfunction. Nat Commun.

[CR39] Kars M, Yang L, Gregor MF, Mohammed BS, Pietka TA, Finck BN, Patterson BW, Horton JD, Mittendorfer B, Hotamisligil GS (2010). Tauroursodeoxycholic acid may improve liver and muscle but not adipose tissue insulin sensitivity in obese men and women. Diabetes.

[CR40] Kelley ST, Skarra DV, Rivera AJ, Thackray VG (2016). The gut microbiome is altered in a letrozole-induced mouse model of polycystic ovary syndrome. PLoS ONE.

[CR41] Kim DM (2015). Gut microbiota-mediated drug-antibiotic interactions. Drug Metab Dispos.

[CR42] Knowler WC, Barrett-Connor E, Fowler SE, Hamman RF, Lachin JM, Walker EA, Nathan DM (2002). Reduction in the incidence of type 2 diabetes with lifestyle intervention or metformin. N Engl J Med.

[CR43] Kovatcheva-Datchary P, Nilsson A, Akrami R, Lee YS, De Vadder F, Arora T, Hallen A, Martens E, Bjorck I, Backhed F (2015). Dietary fiber-induced improvement in glucose metabolism is associated with increased abundance of prevotella. Cell Metab.

[CR44] Kristensen NB, Bryrup T, Allin KH, Nielsen T, Hansen TH, Pedersen O (2016). Alterations in fecal microbiota composition by probiotic supplementation in healthy adults: a systematic review of randomized controlled trials. Genome Med.

[CR45] Larraufie P, Martin-Gallausiaux C, Lapaque N, Dore J, Gribble FM, Reimann F, Blottiere HM (2018). SCFAs strongly stimulate PYY production in human enteroendocrine cells. Sci Rep.

[CR46] Larsson H, Lindgärde F, Berglund G, Ahrén B (2000). Prediction of diabetes using ADA or WHO criteria in post-menopausal women: a 10-year follow-up study. Diabetologia.

[CR47] Laursen MF, Laursen RP, Larnkjær A, Michaelsen KF, Bahl MI, Licht TR (2017). Administration of two probiotic strains during early childhood does not affect the endogenous gut microbiota composition despite probiotic proliferation. BMC Microbiol.

[CR48] Le Roy T, Llopis M, Lepage P, Bruneau A, Rabot S, Bevilacqua C, Martin P, Philippe C, Walker F, Bado A (2013). Intestinal microbiota determines development of non-alcoholic fatty liver disease in mice. Gut.

[CR49] Levy M, Thaiss CA, Zeevi D, Dohnalova L, Zilberman-Schapira G, Mahdi JA, David E, Savidor A, Korem T, Herzig Y (2015). Microbiota-modulated metabolites shape the intestinal microenvironment by regulating NLRP6 inflammasome signaling. Cell.

[CR50] Li GL, Xie C, Lu SY, Nichols RG, Tian Y, Li LC, Patel D, Ma YY, Brocker CN, Yan TT (2017). Intermittent fasting promotes white adipose browning and decreases obesity by shaping the gut microbiota. Cell Metab.

[CR51] Li Z, Yi CX, Katiraei S, Kooijman S, Zhou E, Chung CK, Gao Y, van den Heuvel JK, Meijer OC, Berbee JFP (2018). Butyrate reduces appetite and activates brown adipose tissue via the gut-brain neural circuit. Gut.

[CR52] Lim PS, Loke CF, Ho YW, Tan HY (2020) Cholesterol homeostasis associated with probiotic supplementation in vivo. J Appl Microbiol 10.1111/jam.14678 32356362

[CR53] Lindheim L, Bashir M, Munzker J, Trummer C, Zachhuber V, Leber B, Horvath A, Pieber TR, Gorkiewicz G, Stadlbauer V (2017). Alterations in gut microbiome composition and barrier function are associated with reproductive and metabolic defects in women with polycystic ovary syndrome (PCOS): a pilot study. PLoS ONE.

[CR54] Liu R, Hong J, Xu X, Feng Q, Zhang D, Gu Y, Shi J, Zhao S, Liu W, Wang X (2017). Gut microbiome and serum metabolome alterations in obesity and after weight-loss intervention. Nat Med.

[CR55] Liu R, Zhang C, Shi Y, Zhang F, Li L, Wang X, Ling Y, Fu H, Dong W, Shen J (2017). Dysbiosis of gut microbiota associated with clinical parameters in polycystic ovary syndrome. Front Microbiol.

[CR56] Longo VD, Panda S (2016). Fasting, circadian rhythms, and time-restricted feeding in healthy lifespan. Cell Metab.

[CR57] Louis P, Hold GL, Flint HJ (2014). The gut microbiota, bacterial metabolites and colorectal cancer. Nat Rev Microbiol.

[CR58] Manco M, Putignani L, Bottazzo GF (2010). Gut microbiota, lipopolysaccharides, and innate immunity in the pathogenesis of obesity and cardiovascular risk. Endocr Rev.

[CR59] Mao K, Baptista AP, Tamoutounour S, Zhuang L, Bouladoux N, Martins AJ, Huang Y, Gerner MY, Belkaid Y, Germain RN (2018). Innate and adaptive lymphocytes sequentially shape the gut microbiota and lipid metabolism. Nature.

[CR60] Matsubara T, Li F, Gonzalez FJ (2013). FXR signaling in the enterohepatic system. Mol Cell Endocrinol.

[CR61] Meslier V, Laiola M, Roager HM, De Filippis F, Roume H, Quinquis B, Giacco R, Mennella I, Ferracane R, Pons N (2020). Mediterranean diet intervention in overweight and obese subjects lowers plasma cholesterol and causes changes in the gut microbiome and metabolome independently of energy intake. Gut.

[CR62] Miyamoto J, Igarashi M, Watanabe K, Karaki SI, Mukouyama H, Kishino S, Li X, Ichimura A, Irie J, Sugimoto Y (2019). Gut microbiota confers host resistance to obesity by metabolizing dietary polyunsaturated fatty acids. Nat Commun.

[CR63] Nathan DM, Davidson MB, DeFronzo RA, Heine RJ, Henry RR, Pratley R, Zinman B, American Diabetes A (2007). Impaired fasting glucose and impaired glucose tolerance: implications for care. Diabetes Care.

[CR64] Norman RJ, Dewailly D, Legro RS, Hickey TE (2007). Polycystic ovary syndrome. The Lancet.

[CR65] Perez-Munoz ME, Arrieta MC, Ramer-Tait AE, Walter J (2017). A critical assessment of the “sterile womb” and “in utero colonization” hypotheses: implications for research on the pioneer infant microbiome. Microbiome.

[CR66] Pi Y, Mu C, Gao K, Liu Z, Peng Y, Zhu W (2020). Increasing the hindgut carbohydrate/protein ratio by cecal infusion of corn starch or casein hydrolysate drives gut microbiota-related bile acid metabolism to stimulate colonic barrier function. mSystems.

[CR67] Popkin BM, Adair LS, Ng SW (2012). Global nutrition transition and the pandemic of obesity in developing countries. Nutr Rev.

[CR68] Psichas A, Sleeth ML, Murphy KG, Brooks L, Bewick GA, Hanyaloglu AC, Ghatei MA, Bloom SR, Frost G (2015). The short chain fatty acid propionate stimulates GLP-1 and PYY secretion via free fatty acid receptor 2 in rodents. Int J Obes (Lond).

[CR69] Qi X, Yun C, Sun L, Xia J, Wu Q, Wang Y, Wang L, Zhang Y, Liang X, Wang L (2019). Gut microbiota-bile acid-interleukin-22 axis orchestrates polycystic ovary syndrome. Nat Med.

[CR70] Quinn RA, Melnik AV, Vrbanac A, Fu T, Patras KA, Christy MP, Bodai Z, Belda-Ferre P, Tripathi A, Chung LK (2020). Global chemical effects of the microbiome include new bile-acid conjugations. Nature.

[CR71] Ragsdale SW, Pierce E (2008). Acetogenesis and the Wood-Ljungdahl pathway of CO(2) fixation. Biochem Biophys Acta.

[CR72] Rao RK, Seth A, Sheth P (2004). Recent advances in alcoholic liver disease I. Role of intestinal permeability and endotoxemia in alcoholic liver disease. Am J Physiol Gastrointest Liver Physiol.

[CR73] Reilly SM, Saltiel AR (2017). Adapting to obesity with adipose tissue inflammation. Nat Rev Endocrinol.

[CR74] Rubio LA, Aranda-Olmedo I, Martín-Pedrosa M (2020) Inclusion of limited amounts of extruded legumes plus cereal mixes in normocaloric or obesogenic diets for rats: effects on lipid profile. Foods (Basel, Switzerland) 9 10.3390/foods9060704 PMC7353632 32492801

[CR75] Sanna S, van Zuydam NR, Mahajan A, Kurilshikov A, Vich Vila A, Võsa U, Mujagic Z, Masclee AAM, Jonkers D, Oosting M (2019). Causal relationships among the gut microbiome, short-chain fatty acids and metabolic diseases. Nat Genet.

[CR76] Santaguida PL, Balion C, Hunt D, Morrison K, Gerstein H, Raina P, Booker L, Yazdi H (2005) Diagnosis, prognosis, and treatment of impaired glucose tolerance and impaired fasting glucose. Evidence report/technology assessment (Summary), 1–11 PMC4780988 16194123

[CR77] Schroeder BO, Backhed F (2016). Signals from the gut microbiota to distant organs in physiology and disease. Nat Med.

[CR78] Schwimmer JB, Johnson JS, Angeles JE, Behling C, Belt PH, Borecki I, Bross C, Durelle J, Goyal NP, Hamilton G (2019). Microbiome signatures associated with steatohepatitis and moderate to severe fibrosis in children with nonalcoholic fatty liver disease. Gastroenterology.

[CR79] Sender R, Fuchs S, Milo R (2016). Are we really vastly outnumbered? revisiting the ratio of bacterial to host cells in humans. Cell.

[CR80] Shaw JE, Zimmet PZ, de Courten M, Dowse GK, Chitson P, Gareeboo H, Hemraj F, Fareed D, Tuomilehto J, Alberti KG (1999). Impaired fasting glucose or impaired glucose tolerance. What best predicts future diabetes in Mauritius?. Diabetes Care.

[CR81] Song X, Sun X, Oh SF, Wu M, Zhang Y, Zheng W, Geva-Zatorsky N, Jupp R, Mathis D, Benoist C (2020). Microbial bile acid metabolites modulate gut RORγ(+) regulatory T cell homeostasis. Nature.

[CR82] Sun J, Buys NJ (2016). Glucose- and glycaemic factor-lowering effects of probiotics on diabetes: a meta-analysis of randomised placebo-controlled trials. Br J Nutr.

[CR83] Sun L, Xie C, Wang G, Wu Y, Wu Q, Wang X, Liu J, Deng Y, Xia J, Chen B (2018). Gut microbiota and intestinal FXR mediate the clinical benefits of metformin. Nat Med.

[CR84] Sun L, Pang Y, Wang X, Wu Q, Liu H, Liu B, Liu G, Ye M, Kong W, Jiang C (2019). Ablation of gut microbiota alleviates obesity-induced hepatic steatosis and glucose intolerance by modulating bile acid metabolism in hamsters. Acta Pharm Sin B.

[CR85] Sutton EF, Beyl R, Early KS, Cefalu WT, Ravussin E, Peterson CM (2018). Early time-restricted feeding improves insulin sensitivity, blood pressure, and oxidative stress even without weight loss in men with prediabetes. Cell Metab.

[CR86] Swann JR, Want EJ, Geier FM, Spagou K, Wilson ID, Sidaway JE, Nicholson JK, Holmes E (2011). Systemic gut microbial modulation of bile acid metabolism in host tissue compartments. Proc Natl Acad Sci USA.

[CR87] Thingholm LB, Ruhlemann MC, Koch M, Fuqua B, Laucke G, Boehm R, Bang C, Franzosa EA, Hubenthal M, Rahnavard A (2019). Obese Individuals with and without Type 2 diabetes show different gut microbial functional capacity and composition. Cell Host Microbe.

[CR88] Tolhurst G, Heffron H, Lam YS, Parker HE, Habib AM, Diakogiannaki E, Cameron J, Grosse J, Reimann F, Gribble FM (2012). Short-chain fatty acids stimulate glucagon-like peptide-1 secretion via the G-protein-coupled receptor FFAR2. Diabetes.

[CR89] Trabelsi MS, Daoudi M, Prawitt J, Ducastel S, Touche V, Sayin SI, Perino A, Brighton CA, Sebti Y, Kluza J (2015). Farnesoid X receptor inhibits glucagon-like peptide-1 production by enteroendocrine L cells. Nat Commun.

[CR90] Tuomilehto J, Lindström J, Eriksson JG, Valle TT, Hämäläinen H, Ilanne-Parikka P, Keinänen-Kiukaanniemi S, Laakso M, Louheranta A, Rastas M (2001). Prevention of type 2 diabetes mellitus by changes in lifestyle among subjects with impaired glucose tolerance. N Engl J Med.

[CR91] Turnbaugh PJ, Ley RE, Mahowald MA, Magrini V, Mardis ER, Gordon JI (2006). An obesity-associated gut microbiome with increased capacity for energy harvest. Nature.

[CR92] Turnbaugh PJ, Backhed F, Fulton L, Gordon JI (2008). Diet-induced obesity is linked to marked but reversible alterations in the mouse distal gut microbiome. Cell Host Microbe.

[CR93] Usami M, Kishimoto K, Ohata A, Miyoshi M, Aoyama M, Fueda Y, Kotani J (2008). Butyrate and trichostatin A attenuate nuclear factor kappaB activation and tumor necrosis factor alpha secretion and increase prostaglandin E2 secretion in human peripheral blood mononuclear cells. Nutr Res.

[CR94] Vendrame F, Gottlieb PA (2004). Prediabetes: prediction and prevention trials. Endocrinol Metab Clin N Am.

[CR95] Vernocchi P, Del Chierico F, Putignani L (2016). Gut microbiota profiling: metabolomics based approach to unravel compounds affecting human health. Front Microbiol.

[CR96] Virani SS, Alonso A, Benjamin EJ, Bittencourt MS, Callaway CW, Carson AP, Chamberlain AM, Chang AR, Cheng S, Delling FN (2020). Heart Disease and Stroke Statistics-2020 update: a report from the American Heart Association. Circulation.

[CR97] Wang C, Nagata S, Asahara T, Yuki N, Matsuda K, Tsuji H, Takahashi T, Nomoto K, Yamashiro Y (2015). Intestinal microbiota profiles of healthy pre-school and school-age children and effects of probiotic supplementation. Ann Nutr Metab.

[CR98] Wang B, Jiang X, Cao M, Ge J, Bao Q, Tang L, Chen Y, Li L (2016). Altered fecal microbiota correlates with liver biochemistry in nonobese patients with non-alcoholic fatty liver disease. Sci Rep.

[CR99] Wang L, Ren B, Zhang Q, Chu C, Zhao Z, Wu J, Zhao W, Liu Z, Liu X (2020). Methionine restriction alleviates high-fat diet-induced obesity: involvement of diurnal metabolism of lipids and bile acids. Biochim Biophys Acta.

[CR100] Whang A, Nagpal R, Yadav H (2019). Bi-directional drug-microbiome interactions of anti-diabetics. EbioMedicine.

[CR101] Whitman WB, Coleman DC, Wiebe WJ (1998). Prokaryotes: the unseen majority. Proc Natl Acad Sci USA.

[CR102] Worthmann A, John C, Ruhlemann MC, Baguhl M, Heinsen FA, Schaltenberg N, Heine M, Schlein C, Evangelakos I, Mineo C (2017). Cold-induced conversion of cholesterol to bile acids in mice shapes the gut microbiome and promotes adaptive thermogenesis. Nat Med.

[CR103] Wu GD, Chen J, Hoffmann C, Bittinger K, Chen YY, Keilbaugh SA, Bewtra M, Knights D, Walters WA, Knight R (2011). Linking long-term dietary patterns with gut microbial enterotypes. Science.

[CR104] Wu H, Esteve E, Tremaroli V, Khan MT, Caesar R, Manneras-Holm L, Stahlaman M, Olsson LM, Serino M, Planas-Felix M (2017). Metformin alters the gut microbiome of individuals with treatment-naive type 2 diabetes, contributing to the therapeutic effects of the drug. Nat Med.

[CR105] Xie C, Jiang C, Shi J, Gao X, Sun D, Sun L, Wang T, Takahashi S, Anitha M, Krausz KW (2017). An intestinal farnesoid X receptor-ceramide signaling axis modulates hepatic gluconeogenesis in mice. Diabetes.

[CR106] Yao CK, Muir JG, Gibson PR (2016). Review article: insights into colonic protein fermentation, its modulation and potential health implications. Aliment Pharmacol Ther.

[CR107] Younossi ZM, Koenig AB, Abdelatif D, Fazel Y, Henry L, Wymer M (2016). Global epidemiology of nonalcoholic fatty liver disease-meta-analytic assessment of prevalence, incidence, and outcomes. Hepatology.

[CR108] Zeevi D, Korem T, Zmora N, Israeli D, Rothschild D, Weinberger A, Ben-Yacov O, Lador D, Avnit-Sagi T, Lotan-Pompan M (2015). Personalized nutrition by prediction of glycemic responses. Cell.

[CR109] Zelante T, Iannitti Rossana G, Cunha C, De Luca A, Giovannini G, Pieraccini G, Zecchi R, D’Angelo C, Massi-Benedetti C, Fallarino F (2013). Tryptophan catabolites from microbiota engage aryl hydrocarbon receptor and balance mucosal reactivity via interleukin-22. Immunity.

[CR110] Zhang Q, Li Y, Chen L (2015). Effect of berberine in treating type 2 diabetes mellitus and complications and its relevant mechanisms. China J Chin Mater Med.

[CR111] Zhang L, Xie C, Nichols RG, Chan SH, Jiang C, Hao R, Smith PB, Cai J, Simons MN, Hatzakis E (2016). Farnesoid X receptor signaling shapes the gut microbiota and controls hepatic lipid metabolism. mSystems.

[CR112] Zhang W, Xu JH, Yu T, Chen QK (2019). Effects of berberine and metformin on intestinal inflammation and gut microbiome composition in db/db mice. Biomed Pharmacother.

[CR113] Zhang X, Zhang Y, Wang P, Zhang SY, Dong Y, Zeng G, Yan Y, Sun L, Wu Q, Liu H (2019). Adipocyte hypoxia-inducible factor 2alpha suppresses atherosclerosis by promoting adipose ceramide catabolism. Cell Metab.

[CR114] Zhang Z, Zhou H, Zhou X, Sun J, Liang X, Lv Y, Bai L, Zhang J, Gong P, Liu T (2020). Lactobacillus casei YRL577 ameliorates markers of non-alcoholic fatty liver and alters expression of genes within the intestinal bile acid pathway. Br J Nutr.

[CR115] Zheng Y, Ley SH, Hu FB (2018). Global aetiology and epidemiology of type 2 diabetes mellitus and its complications. Nat Rev Endocrinol.

[CR116] Zhu L, Baker SS, Gill C, Liu W, Alkhouri R, Baker RD, Gill SR (2013). Characterization of gut microbiomes in nonalcoholic steatohepatitis (NASH) patients: a connection between endogenous alcohol and NASH. Hepatology.

